# The profile of HDL-C subfractions and their association with cardiovascular risk in the Hungarian general and Roma populations

**DOI:** 10.1038/s41598-022-15192-9

**Published:** 2022-06-28

**Authors:** Peter Piko, Zsigmond Kosa, Janos Sandor, Ildiko Seres, Gyorgy Paragh, Roza Adany

**Affiliations:** 1 ELKH-DE Public Health Research Group, University of Debrecen, Debrecen, 4028 Hungary; 2 Department of Health Methodology and Public Health, Faculty of Health, University of Debrecen, Nyíregyháza, 4400 Hungary; 3 Department of Public Health and Epidemiology, Faculty of Medicine, University of Debrecen, Debrecen, 4028 Hungary; 4 Department of Internal Medicine, Division of Metabolic Disorders, Faculty of Medicine, University of Debrecen, Debrecen, 4032 Hungary

**Keywords:** Biomarkers, Cardiology, Risk factors

## Abstract

High-density lipoprotein cholesterol (HDL-C) is not a homogenous lipid fraction, but it can be further divided into subfractions. It is well-known that the Roma population has a high prevalence of reduced HDL-C levels and cardiovascular diseases (CVDs). However, it is unknown how this reduction affects different HDL subfractions, and whether changes in their quantity/representation are associated with an increased cardiovascular risk among them. In the present study, the HDL subfraction profile of the Hungarian general (HG) and the Roma populations were compared, and the subfractions showing a significant difference between the two populations were identified. The association of HDL subfractions with CVD risk estimated by the Framingham risk score (FRS) and the Systematic COronary Risk Evaluation (SCORE) algorithms were also defined. The present study is the first to find a significant association between HDL subfractions and cardiovascular risk estimated by FRS and SCORE. Ten HDL subfractions were investigated on small but carefully selected samples comprising 100 control subjects (with normal lipid profile) and 277 case subjects (with reduced HDL-C levels) from HG and Roma populations of a complex health survey. The level of HDL-1 to 3 subfractions and HDL-L showed a significant inverse association with cardiovascular risk estimated by both SCORE and FRS algorithms, whereas HDL-4 to 6 and HDL-I only for FRS. A higher representation (in %) of HDL-1 to 3 has a significant risk-reducing effect, while HDL-8 to 10 has a risk-increasing effect estimated by FRS. Our results confirmed that reduced levels of HDL-6 and -7 expressed in mmol/L were significantly associated with Roma ethnicity.

## Introduction

In 2019, an estimated 17.9 million people died from cardiovascular diseases (CVDs), representing 32% of all global deaths and making it the leading cause of death worldwide (85% of which were due to stroke or heart attack)^1^. CVDs were also responsible for 38% of the 17 million premature (under 70 years) deaths due to non-communicable diseases in the same year. The development of these diseases can be prevented by addressing lifestyle and environmental risk factors such as unhealthy diet and obesity, harmful alcohol consumption, smoking, and the lack of physical activity^1^. In addition to these changeable risk factors, unchangeable ones, such as age, sex, race, and genetic predisposition, also have a significant effect on an individual's cardiovascular risk^2^.

Numerous cardiovascular risk estimation algorithms are known which use changeable and unchangeable factors to estimate the probability of a cardiovascular event within a defined period^3^. The best known and most widely used of these algorithms are the Framingham Risk Score (FRS)^4^ and the Systematic COronary Risk Evaluation (SCORE) calculations^5^. Both models estimate the risk of various cardiovascular events within 10 years and consider an individual's total cholesterol (TC) and high-density lipoprotein cholesterol levels (HDL-C), among many other CVD risk factors.

The lipid profile including TC, low-density lipoprotein cholesterol (LDL-C), HDL-C, and triglyceride (TG) is strongly associated with the risk of developing CVDs^6^. Concerning the lipid metabolic pathways, the serum levels of these lipids are highly correlated with each other, and a stronger association is obtained when their effects on cardiovascular risk are considered together. Epidemiological studies have shown that high levels of LDL-C and TG combined with low levels of HDL-C are associated with an increased risk for the development of CVDs^6^ and can, therefore, be used as a risk predictor, as well as to detect cardiovascular diseases earlier and treat them more effectively.

People on low incomes often have less access to preventive services and do not benefit from primary health care programs aimed at early detection and treatment of CVDs. As a result, these people are frequently diagnosed late and die at a younger age from CVDs or other non-communicable diseases, often in their most productive years^1,7^. Moreover, in addition to individual health risks, at a macroeconomic level, CVDs impose a heavy burden on the economy of several countries^1,8,9^.

The socio-economic situation of the Roma population is significantly less favorable compared to general ones regardless of their host country^10–14^. Evidence shows that the life expectancy at birth of the Roma population is significantly lower than that of non-Roma populations^15^. The prevalence of cardiovascular risk factors^16–20^ and, thus, the overall estimated cardiovascular risk are significantly higher among the Roma than the host country average^21^. Previous studies have shown that reduced HDL-C levels are very common in the Roma population^22–25^ and that the estimated 10-year cardiovascular risk is also significantly higher in the case of both sexes among them than in the Hungarian general population^21^.

It is a well-known fact that elevated HDL-C levels in the blood show an inverse relationship with the incidence of CVDs^26^ and in addition to being involved in the mechanism of reverse cholesterol transport, it is also known to have several beneficial health effects (anti-thrombotic^27^, anti-inflammatory^28^, anti-oxidative^29^, and pro-vasodilatory^30^).

In recent years, our research team has found that genetic factors, in addition to lifestyle and environmental ones, play a very important role in the high prevalence of reduced HDL-C levels among the Roma^31–34^. In the interpretation of the high frequency of low HDL-C levels among the Roma, it has to be also considered that HDL-C particles do not show a uniform pattern; HDL can be further divided into subfractions which differ in size, density, and components and have different effects on cardiovascular risk^35–39^. HDL subfractions are classified into three major subclasses: large HDLs (HDL-L), intermediate HDLs (HDL-I), and small HDLs (HDL-S). A higher concentration of large HDL-C but not that of intermediate, small, or total HDL-C is associated with lower cardiovascular risk^37,38^, whereas the HDL-S shows a relationship with an elevated risk for CVDs^40^.

In a single study published in 2018 by Hubková and her colleagues, the serum lipoprotein profiles of Roma and non-Roma populations from Slovakia were compared and the concentrations of the small HDL subfractions (8 to 10 by Lipoprint test) were described as significantly lower among the Roma^41^. However, whether this phenomenon is a unique feature of the Roma population, or a general characteristic consequence of the reduced HDL-C level found very frequently among them was not investigated. Furthermore, the reduced HDL-S level (and the associated reduced cardiovascular risk) contradicts the numerous publications describing an increased cardiovascular risk among the Roma in general^16–21^.

Currently, there is only a limited number of publications that describe the association of HDL subfractions with cardiovascular risk estimated by algorithms^37,42^. What is more, no study has been published so far on the association of the SCORE risk assessment, the most widely used cardiovascular risk algorithm in Europe, with HDL subfraction profile.

Therefore, the major goal of our study is to try and answer the following questions: (1) Is there a difference in the quantity (in mmol/L) or representation (in %) of HDL subfractions between Hungarian general and Roma individuals? (2) Is there a correlation between the cardiovascular risk estimated by SCORE or FRS and the profile of HDL subfractions?

## Material and methods

### Study design and populations

Study design and data collection were described in detail in our previous study^43^. In brief, a complex health survey was designed and performed to create a database for comparative and association studies to better understand the background of the very unfavorable health of Roma people in comparison with the Hungarian general population, with a special emphasis on the high burden of cardiometabolic diseases. This cross-sectional study had three main pillars including questionnaire-based, physical, and laboratory investigations involving adults aged 20–64 years from the Hungarian general (HG) and Hungarian Roma (Roma) populations. Altogether, 832 participants were recruited for the study including 417 HG (185 male and 232 female) and 415 Roma (108 male and 307 female) subjects. In addition to anthropometric (to define BMI), demographic (sex, age), socioeconomic, and health-related data (among them blood pressure measurement, use of antihypertensive medication, diabetes diagnosed), fasting blood samples were also collected for routine laboratory tests (among them TC, LDL-C, HDL-C, TG, apolipoprotein A1 (ApoAI) and apolipoprotein B (ApoB)). Using the routine laboratory data TG/HDL-C and ApoB/ApoAI ratios were also calculated.

**Figure 1:**
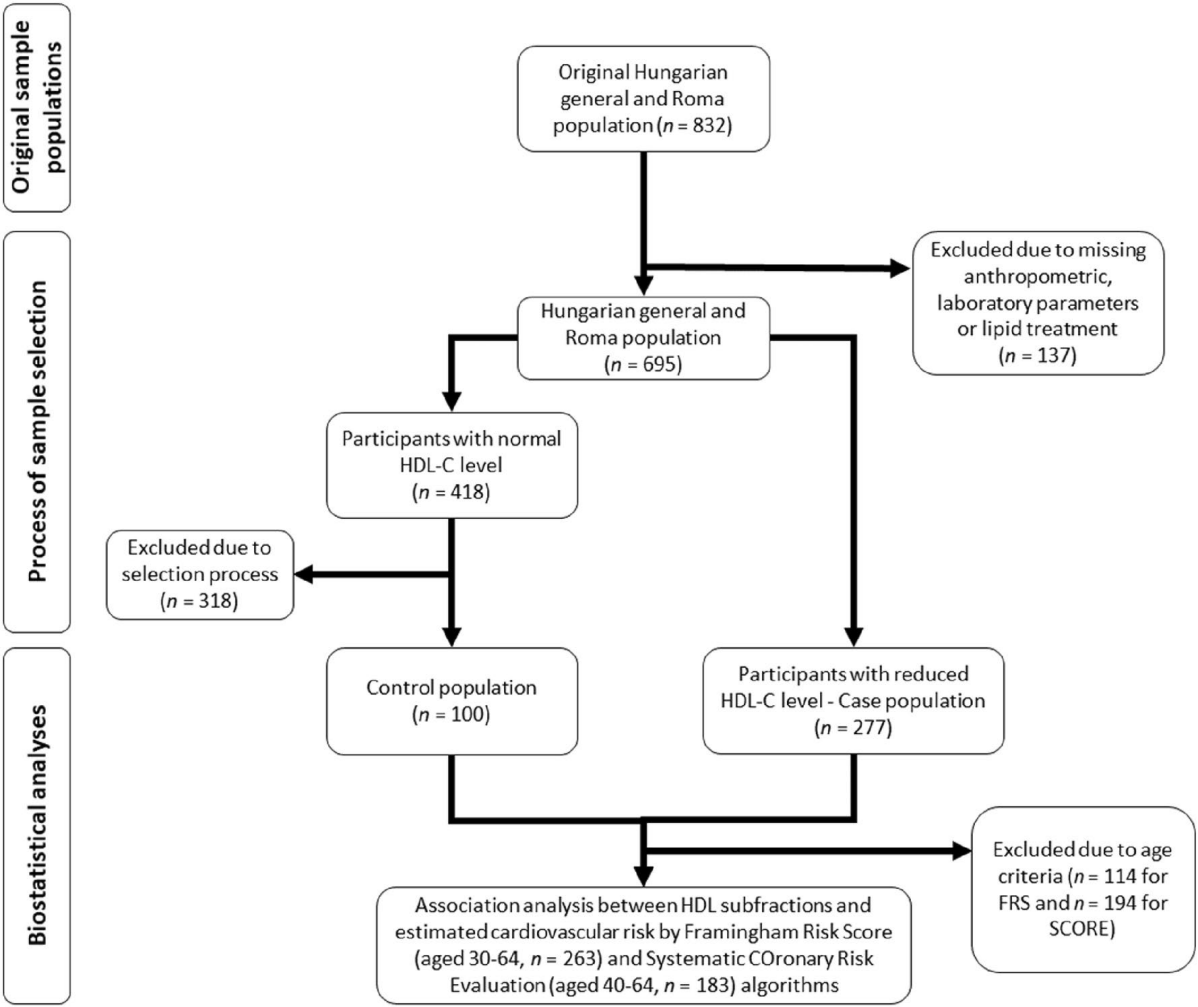
Flowchart showing the process of sample selection and biostatistical analyses.

Participants whose anthropometric and/or laboratory parameters had been missing (20 HG and 47 Roma) and those who had received any lipid treatment (27 HG and 43 Roma) were excluded from further analysis. The remaining 695 persons (370 HG and 325 Roma) were divided into two groups based on their HDL-C levels. The first group included people who had normal HDL-C levels (≥ 1.03 mmol/L in males and ≥ 1.29 mmol/L in females) and had no other lipid abnormality (126 HG and 87 Roma). One hundred persons (25 HG male, 25 Roma male, 25 HG female, and 25 Roma female) were randomly selected from among them to represent the control group of the present study. The second group included people who had reduced HDL-C levels (115 HG and 162 Roma). To examine the association of HDL subfractions with the estimated risk of developing CVDs within 10 years, individuals belonging to the age group defined to the calculation of FRS (30–75 years; 124 HG and 139 Roma) and SCORE (40–65 years; 89 HG and 94 Roma) were studied. For more details, see Fig. 1.

### Analysis of HDL subfractions

HDL is a highly heterogeneous class of lipoproteins that are considered and discussed as a group based solely on the hydrated density of its particles. Several methods are known to subfractionate HDL into subfractions. Most published prospective and clinical studies evaluating the use of HDL subfractions to predict results have used one of the proprietary laboratory tests or proprietary in-house systems available to clinicians: Lipoprint HDL® (gel electrophoresis), Cardio IQ® (ion mobility), NMR LipoProfile® (nuclear magnetic resonance) and, until recently, Vertical auto profile (VAP) ® (ultracentrifugation).

For the present study, HDL subfractions were determined using an electrophoretic method on polyacrylamide gel with the Lipoprint HDL Subfractions Test (Quantimetrix Corp., CA, USA) according to the manufacturer’s instructions. This commercially available test based on the linear polyacrylamide gel electrophoresis method separates and quantifies up to 10 HDL subfractions in serum or plasma.

Concisely, 25 μL serum was added to the 3% polyacrylamide gel tubes along with a 300 μL Lipoprint HDL Loading Gel solution. The tubes contained Sudan Black as a lipophilic dye and were photopolymerized at room temperature for 30 min. Electrophoresis with tubes containing sera samples and the manufacturer’s quality controls were performed at a constant of 3 mA/tube for 50 min. Subfraction bands were identified by their mobility (Rf) using very-LDL (VLDL) + LDL as the starting (Rf 0.0) and albumin as the ending (Rf 1.0) reference point and were scanned with an ArtixScan M1 digital scanner (Microtek International Inc., CA, USA).

Ten HDL subfractions were differentiated between VLDL + LDL and albumin peaks and were grouped into three major classes: HDL-L (from HDL-1 to 3), HDL-I (from HDL-4 to 7), and HDL-S (from HDL-8 to 10) HDL subfractions. Cholesterol concentrations of the HDL particle subsets were calculated with Lipoware software (Quantimetrix Corp., CA, USA) by multiplying the total cholesterol concentration of the samples by the relative area under the curve of the subfraction bands.

### Estimation of the cardiovascular risk by FRS and SCORE in Study Populations

In the present study, we estimated the cardiovascular risk of HG and Roma populations by applying the two most used risk estimation models (FRS and SCORE) in Europe. Both algorithms are sex-specific and estimate the risk of a cardiovascular event occurring within 10 years.

The first version of FRS was developed based on data obtained from the Framingham Heart Study, to estimate the 10-year risk of developing a coronary heart disease, which was later revised to calculate also the 10-year risk of developing CVDs in general. In the present study, we used both versions of the FRS developed for hard coronary heart disease (FRS_CHD_)^44^ and cardiovascular disease (FRS_CVD_)^4^. Both versions consider age, sex, total cholesterol, HDL-C levels, systolic blood pressure, high blood pressure treatment, and smoking status; for FRS_CVD_, addition, diabetes status is also included in the algorithm. Analyses for FRS were performed on participants of the study populations aged 30–64.

An estimated risk based on SCORE, which is the algorithm recommended by the 2007 European Society of Cardiology guidelines on cardiovascular disease prevention in clinical practice—was also calculated for both study populations^5^. The model has been calibrated according to each European country’s mortality statistics. In the present study, the SCORE algorithm used for countries in the high-risk group was applied to both study populations. All analyses for SCORE were performed for participants of the study populations aged 40–64.

A more detailed explanation of the cardiovascular risk models used in the present study is described in our previous publication^21^.

### Statistical analyses

All statistical analyses were conducted by using SPSS (version 26) software (IBM Company, Armonk, NY, USA). Prevalence data were compared by the χ^2^ test. Comparisons between subgroups were performed by Student’s unpaired t-test in case of normally distributed variables and by Mann–Whitney U-test in case of variables with non-normal distribution. Correlations between continuous variables were assessed by linear regression analysis, while logistic regression was used for binary outcome variables. Bonferroni correction was applied for multiple analyses of the same dependent variable to avoid type I error and the *p* value determined by Bonferroni correction were considered as the threshold for statistical significance.

### Ethics declarations

All subjects gave their informed consent for inclusion before they participated in the study. The study was conducted following the Declaration of Helsinki, and the protocol was approved by the Ethics Committee of the Hungarian Scientific Council on Health (61327-2017/EKU).

## Results

### Characteristics of study populations by factors used to estimate cardiovascular risk and lipid profile of the study populations by HDL-C status

When examining the characteristics of the study populations with a normal HDL-C level, a significant difference between the Roma and the HG population was observed in the prevalence of current smokers (HG: 32.00% vs. 62.22%, *p* = 0.004).

In the group with reduced HDL-C levels, there was a significant difference in systolic blood pressure (HG: 127.90 mmHg vs. Roma: 119.75 mmHg, *p* < 0.001), distribution of sex (36.52% male/63.48% female in the HG vs. 20.37% male/79.63% female in the Roma; *p* = 0.003) and the prevalence of current smokers (HG: 39.13% vs. Roma 70.19%, *p* < 0.001) between the study populations. See Supplementary Table 1 for more details.

For the groups with normal and reduced HDL-C levels, there were no significant differences in either lipid or apoprotein profiles between the study populations. See Table 1 for more details.

**Table 1:** Lipid and apolipoprotein profiles of study populations by HDL-C status (normal vs. reduced HDL-C levels).

	Normal HDL-C levels			Reduced HDL-C levels		
	Hungarian general (n = 50)	Roma (n = 50)	*p* value	Hungarian general (n = 115)	Roma (n = 162)	*p* value
	Mean (95% CI)			Mean (95% CI)		
HDL-C (mmol/L)	1.62 (1.53–1.71)	1.59 (1.49–1.70)	0.290	1.02 (0.99–1.05)	1.01 (0.98–1.03)	0.678
LDL-C (mmol/L)	2.50 (2.33–2.67)	2.63 (2.50–2.77)	0.359	3.04 (2.86–3.23)	3.17 (3.02–3.32)	0.186
TG (mmol/L)	0.90 (0.80–0.99)	0.88 (0.80–0.96)	0.814	2.06 (1.84–2.28)	1.88 (1.71–2.06)	0.169
TG/HDL-C ratio	0.58 (0.51–0.66)	0.59 (0.52–0.67)	0.697	2.18 (1.89–2.46)	2.00 (1.79–2.22)	0.290
Total cholesterol (mmol/L)	4.39 (4.19–4.59)	4.43 (4.26–4.60)	0.871	4.79 (4.58–5.00)	4.80 (4.63–4.97)	0.978
ApoAI (g/L)	1.66 (1.58–1.74)	1.62 (1.55–1.69)	0.317	1.30 (1.27–1.33)	1.27 (1.25–1.30)	0.109
ApoB (g/L)	0.83 (0.77–0.88)	0.87 (0.83–0.91)	0.257	1.08 (1.03–1.14)	1.12 (1.07–1.17)	0.242
ApoB/ApoAI ratio	0.51 (0.47–0.55)	0.55 (0.51–0.58)	0.094	0.84 (0.80–0.88)	0.89 (0.85–0.93)	0.097

Threshold of significance after Bonferroni correction: *p* < 0.006.

95% CI, 95% confidence interval; HDL-C, high-density lipoprotein cholesterol; LDL-C, low-density lipoprotein; TG, triglyceride; ApoAI, Apolipoprotein A1; ApoB, Apolipoprotein B.

### Comparative analysis of the HDL-C subfraction profiles of the study populations by HDL-C status

**Figure 2:**
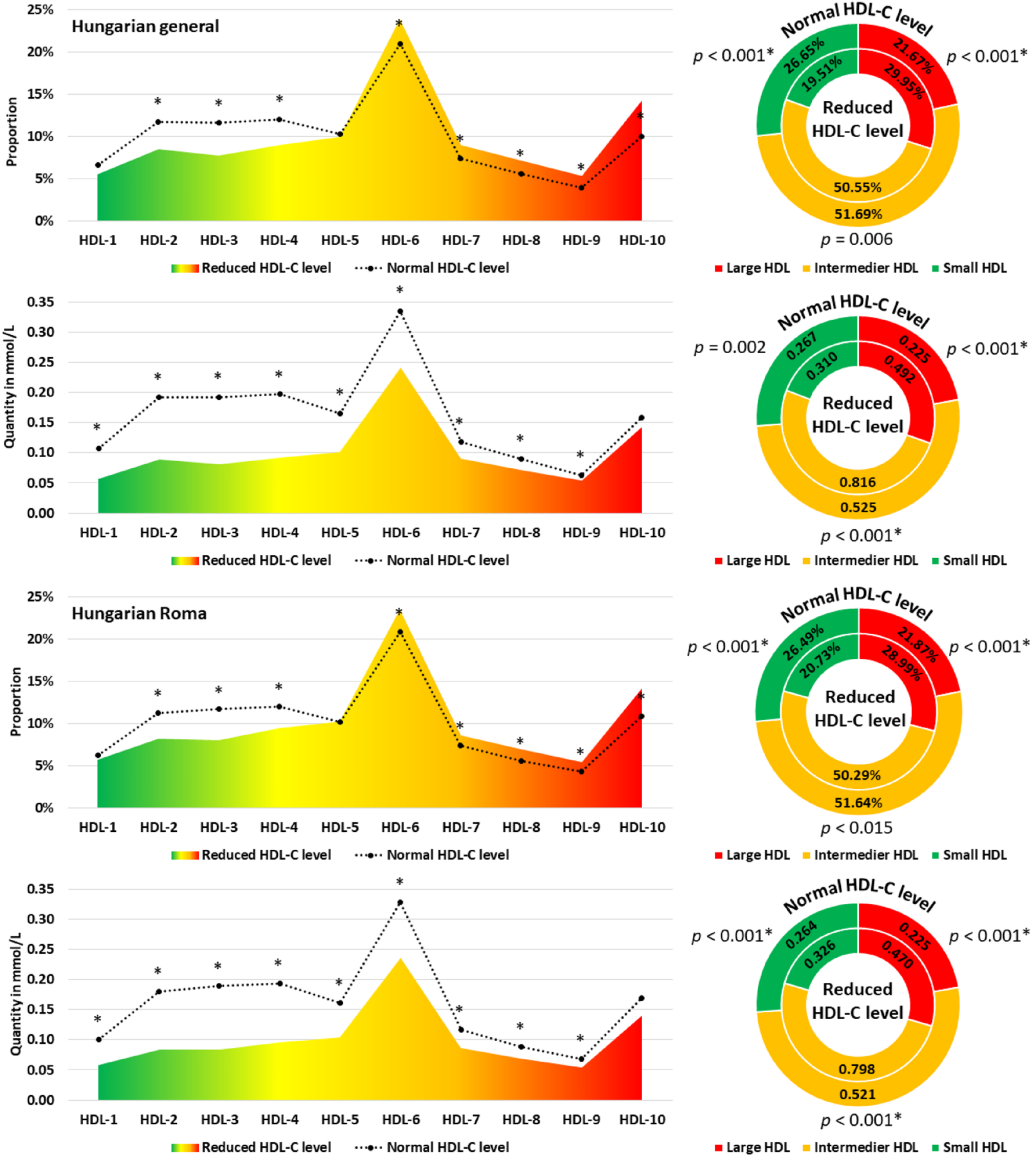
Composition of the HDL subfraction profile (in mmol/L and proportion in %) by HDL-C status (normal and reduced) in the Hungarian general and Roma populations. Large HDL: from HDL-1 to 3; intermediate HDL: from HDL-4 to 7; small HDL: from HDL-8 to 10. *Significant results after test correction (*p* < 0.002).

We compared the HDL subfraction profiles (quantity in mmol/L and proportion in %) of individuals with reduced and normal HDL-C levels. For both populations, individuals with reduced HDL-C levels showed a significant reduction in the proportion of HDL-2 to 4 and an increase in the proportion of HDL-6 to 10. The proportion of HDL-5 remained unchanged. The proportion of HDL-L showed a significant increase while HDL-S showed a decrease in those with reduced HDL-C levels, regardless of the population studied. The proportion of HDL-L remained unchanged. The concentration of HDL-1 to 9 subfractions was significantly lower in participants with reduced HDL-C status than in those with normal HDL-C status in both populations. See Fig. 2 for more details.

There is no difference in the HDL subfraction profiles (neither mmol/L nor proportion in %) between those with normal or reduced HDL-C levels in the two study populations. In both cases, HDL-6 is the most predominant while HDL-9 is the least predominant subfraction. Regardless of the examined population and the HDL-C status, the HDL-I subclass was the most prevalent one. See Supplementary Fig. 1 for more details.

### The effect of Roma ethnicity on HDL subfraction levels (in mmol/L) and proportions (in %) compared to the Hungarian general one

Linear regression analyses were applied to investigate the HDL subfraction profile (as a dependent variable in mmol/L and as a proportion in %) between the HG and the Roma populations. All analyses were adjusted for the independent variable as ethnicity (HG used as reference), sex, age (in years), LDL, and TG levels (in mmol/L).

The results of statistical analyses showed that the Roma population had significantly lower HDL-6 and -7 subfractions in mmol/L compared to the Hungarian general one. In addition, Roma ethnicity was associated with a decrease in all HDL subfractions (although not in all cases significantly). There was no significant effect of Roma ethnicity on the proportion of HDL subfractions. See Table 2 for more details.

**Table 2:** Effect of Roma ethnicity on HDL subfractions (A—in mmol/L and B—in %) compared to the Hungarian general one.

	*β*	95% CI	*p* value
**A**			
HDL-1	− 0.003	− 0.008 to 0.003	0.342
HDL-2	− 0.012	− 0.021 to − 0.003	0.009
HDL-3	− 0.005	− 0.015 to 0.005	0.324
HDL-4	− 0.003	− 0.012 to − 0.006	0.550
HDL-5	− 0.003	− 0.009 to 0.003	0.296
HDL-6	− 0.016	− 0.027 to − 0.006	0.003*
HDL-7	− 0.008	− 0.013 to − 0.003	0.002*
HDL-8	− 0.006	− 0.010 to − 0.002	0.007
HDL-9	< 0.001	− 0.003 to 0.003	0.884
HDL-10	− 0.003	− 0.013 to 0.006	0.502
HDL-L	− 0.020	− 0.041 to 0.001	0.066
HDL-I	− 0.032	− 0.059 to − 0.005	0.022
HDL-S	− 0.008	− 0.023 to 0.007	0.290
**B**			
HDL-1	0.027	− 0.384 to 0.439	0.896
HDL-2	− 0.513	− 0.957 to − 0.069	0.024
HDL-3	0.131	− 0.344 to 0.606	0.589
HDL-4	0.190	− 0.214 to 0.594	0.355
HDL-5	0.200	− 0.044 to 0.443	0.108
HDL-6	− 0.339	− 0.788 to 0.110	0.139
HDL-7	− 0.204	− 0.466 to 0.058	0.127
HDL-8	− 0.149	− 0.386 to 0.088	0.218
HDL-9	0.193	− 0.010 to 0.397	0.063
HDL-10	0.333	− 0.394 to 1.060	0.368
HDL-L	− 0.416	− 1.419 to 0.587	0.415
HDL-I	− 0.047	− 0.682 to 0.588	0.884
HDL-S	0.418	− 0.594 to 1.430	0.417

HDL-L, large HDL (from HDL-1 to 3); HDL-I, intermediate HDL (from HDL-4 to 7); HDL-S, small HDL (from HDL-8 to 10).

*Significant results after test correction (*p* < 0.004).

### Association of HDL subfractions with the 10-year risk of CVD events estimated by the FRS and the SCORE algorithms

Linear regression analyses (adjusted for ethnicity) were applied to examine the association between HDL subfractions and the estimated 10-year cardiovascular risk by SCORE and FRS algorithms.

Out of the 10 HDL subfractions examined, increasing levels (in mmol/L) of HDL-1 to 3 subfractions and HDL-L showed significant association with reduced cardiovascular risk estimated by SCORE. The HDL-1 to 6 subfractions as well as HDL-L and HDL-I levels were significantly associated with reduced cardiovascular risk estimated by FRS_CHD_ and FRS_CVD_.

The proportion of HDL subfractions showed no significant correlation with the cardiovascular risk estimated by SCORE. The proportion of HDL-1 to 3 subfractions showed a significant association with reduced cardiovascular risk estimated by the FRS, whereas HDL-6 to 10 subfractions showed an association with an increased cardiovascular risk. The percentage of HDL-4 was only significantly associated with reduced risk estimated by FRS_CHD_. A higher proportion of HDL-L subfractions was associated with a significantly decreased cardiovascular risk, whereas HDL-S was associated with an increased cardiovascular risk estimated by FRS. See Table 3. for more details.

**Table 3:** Effect of HDL subfractions (A—in mmol/L; B—in %) on the estimated cardiovascular risk by Systematic Coronary Risk Evaluation and Framingham Risk Scores.

	Systematic Coronary Risk Evaluation	Framingham Risk Scores	
	High-risk algorithm	CHD	CVD in general
**A**			
HDL-1	− 11.26*	− 28.37*	− 53.98*
HDL-2	− 6.27*	− 13.43*	− 24.99*
HDL-3	− 5.95*	− 12.99*	− 23.85*
HDL-4	− 4.84	− 12.76*	− 23.23*
HDL-5	− 8.50	− 20.06*	− 39.80*
HDL-6	− 5.10	− 9.93*	− 19.9*
HDL-7	− 8.85	− 12.46	− 25.98
HDL-8	− 8.61	− 4.43	− 14.83
HDL-9	− 6.17	11.85	10.57
HDL-10	0.52	7.31	7.93
HDL-L	− 2.65*	− 5.94*	− 10.94*
HDL-I	− 1.88	− 4.32*	− 8.32*
HDL-S	− 0.81	2.48	1.84
**B**			
HDL-1	− 0.13	− 0.40*	− 0.71*
HDL-2	− 0.08	− 0.24*	− 0.41*
HDL-3	− 0.10	− 0.26*	− 0.42*
HDL-4	− 0.10	− 0.29*	− 0.45
HDL-5	0.03	− 0.18	− 0.39
HDL-6	0.04	0.18	0.33
HDL-7	0.09	0.37	0.68
HDL-8	0.13	0.54*	0.92*
HDL-9	0.17	0.74*	1.20*
HDL-10	0.07	0.18*	0.29*
HDL-L	− 0.05	− 0.12*	− 0.20*
HDL-I	0.01	0.02	0.06
HDL-S	0.04	0.13*	0.20*

HDL-L, large HDL (from HDL-1 to 3); HDL-I, intermediate HDL (from HDL-4 to 7); HDL-S, small HDL (from HDL-8 to 10); CVD, cardiovascular diseases; CHD: coronary heart disease.

*Significant results after test correction (*p* < 0.004).

**Figure 3.**
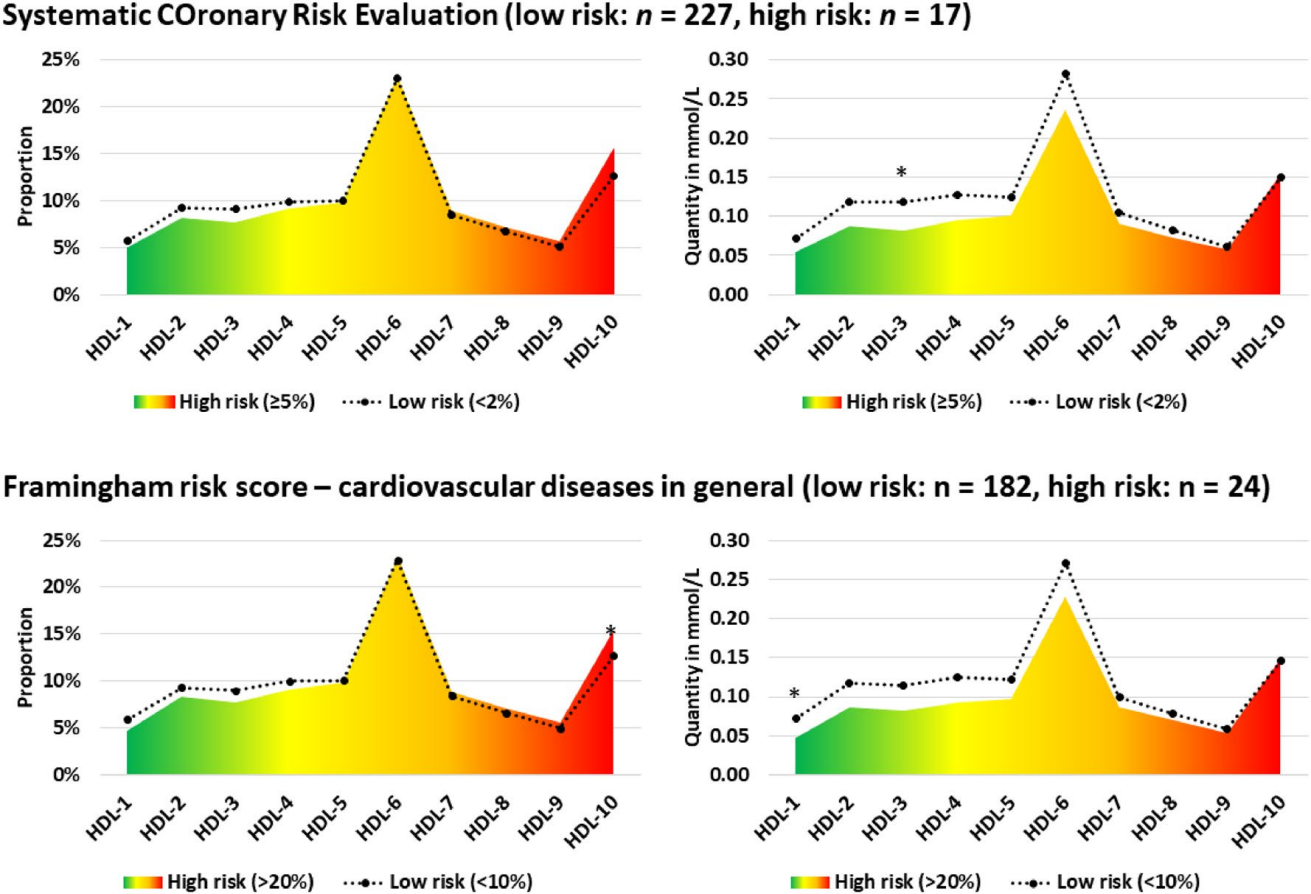
Composition of the HDL subfraction profile (proportion in % and in mmol/L) by low- and high-risk status according to Systematic Coronary Risk Evaluation and Framingham risk score—cardiovascular diseases in general. *Significant results after test correction (*p* < 0.0013).

When comparing groups with low (< 2%) and high (≥ 5%) cardiovascular risk according to SCORE, no significant difference in the proportion of HDL subfractions was observed. The HDL-1 to 7 subfractions in mmol/L were significantly lower in the high-risk group compared with the low-risk group. The proportion of HDL subfractions did not differ significantly between the low (< 10%) and high (> 20%) cardiovascular risk groups based on FRS_CVD_. HDL-1, -4, and -5 subfractions in mmol/L were significantly lower in the high-risk group compared with the low-risk group. For FRS_CHD_, due to the low number of high-risk samples (*n* = 3), the analysis cannot be performed. See Fig. 3. for more details.

Trend analysis showed no significant association between HDL subfractions in mmol/L or % and cardiovascular categories in SCORE. A significant negative trend was measured for HDL-1 to 4, HDL-L, and -I in mmol/L among FRS_CHD_ risk groups. Based on the analysis of the % proportions, HDL-1 to 3 and HDL-L showed a significantly negative trend, while HDL-9, -10, and HDL-S showed a significantly positive trend with the cardiovascular risk estimated by FRS_CHD_. Decreases in HDL-1, -3, -4, and HDL-L and -I subfractions were significantly associated with the increasing trend in cardiovascular risk estimated by FRSCVD. HDL-1 in % was the only one that showed a significant association with the risk estimated by FRSCVD. See Supplementary Tables 2 and 3 for more details.

## Discussion

Recent guidelines for the treatment of dyslipidemia and CVDs focus mainly on LDL and do not include recommendations to increase HDL-C levels^45^. However, both the European SCORE risk calculator and the Framingham Risk Score calculator take HDL-C levels into account when assessing CVD risk and consistently show that increasing HDL-C levels (regardless of the subfraction profile) reduces the risk of developing CVDs in the future.

The present study is the first to investigate and compare the HDL subfraction profiles of the Hungarian general and Roma populations and the association of the HDL profile (based on ten subfractions) with the 10-year risk of CVD events estimated by the FRS and the SCORE algorithms. Although the sample size seems small, it is not significantly different from previous studies using the same methodology^37,46–49^. Identification and quantification of HDL subfractions need an expensive and time-consuming method, so careful identification of subjects who are included in the sample(s) is a crucial requirement in general. In our study, both the control samples and cases were selected from a sample of a complex survey with 832 subjects after careful evaluation of the results published previously^43^.

The HDL subfraction profiles of Roma and Hungarian general populations with the same HDL-C status (normal vs. reduced) were not significantly different suggesting that the reduced HDL-C status is associated with similar subfraction changes in both groups. The results of multi-adjusted linear regression analyses confirmed that significantly reduced levels of HDL-6 and -7 were associated with Roma ethnicity and all other subfractions were also lower than in the Hungarian general population, although not significantly. These results are not in full harmony with the findings of Hubková et al.^41^, who described reduced levels of HDL subfractions 8 to 10 (HDL-S) in Roma compared to the majority of the Slovak population. However, their analyses were based on the result of descriptive statistical analyses and did not adjust for other factors (such as age, sex, and other lipid parameters).

Continuing the analysis, we investigated how the cardiovascular risk estimated by the two most widely used risk algorithms in clinical practice (SCORE and FRS) are associated with changes in the HDL subfraction profile. HDL-1 to -3 and HDL-L in mmol/L showed an inverse significant association with SCORE-estimated cardiovascular risk. Elevated levels of HDL-1 to 6 and HDL-L and HDL-I subfractions expressed in mmol/L showed an inverse association with the FRS estimated 10-year risk of cardiovascular events.

HDL subfractions expressed in % showed no significant association with SCORE-estimated risk. HDL-1 to 3 subfractions and HDL-L expressed as a percentage had an FRS-estimated cardiovascular risk-reducing effect, whereas HDL-8 to 10 and HDL-S had an FRS-estimated cardiovascular risk-increasing effect.

Several methods are available for HDL separation, which are used with varying frequency in studies (gel electrophoresis^49^, nuclear magnetic resonance^50^, and ion mobility^51^). They are based on different physicochemical characteristics of the HDL molecules and separate the particles according to size, charge, density, etc. "Same" HDL subfractions isolated by different separation methods may contain HDL particles that differ greatly in their properties^52^. This phenomenon makes it considerably more difficult or even impossible to compare studies using different separation methods. Therefore, we aim to compare our present results with published results that have used the Lipoprint HDL® platform.

Currently, there is only one article^49^ known to have described the association between HDL subfractions and cardiovascular risk estimated by FRS. This study showed, similar to the present one, that a decrease in the representation of HDL-L is associated with a higher cardiovascular risk, but the association was not statistically significant. There are no known studies that have examined the association between SCORE-estimated cardiovascular risk and HDL subfraction profile. However, several studies have examined the association between HDL subfractions and cardiovascular events.

Goliasch and colleagues found that HDL-L showed an inverse association (OR = 0.48, 95% CI 0.31–0.74, *p* = 0.001), whereas HDL-I was a risk factor (OR = 1.81, 95% CI 1.26–2.60, *p* = 0.001) for myocardial infarction in people aged 40 years and younger^46^. Zhang and colleagues found a significant negative association between the prevalence and incidence of hypertension and the level and proportion of HDL-L, and a positive association between the level and proportion of HDL-S^47^. Xu and colleagues showed that the HDL-L levels and the percentage were significantly lower, while the HDL-S levels and percentage were significantly higher in the coronary artery disease (CAD) group compared with those in the non-CAD group^48^. Li et al. demonstrated that high HDL-L levels were significantly (*p* < 0.05) negatively associated with coronary artery severity as assessed by SYNTAX and Gensini scores. Furthermore, using a log-rank test, they demonstrated that there was a significant (*p* = 0.013) difference between high and low HDL-L subfraction groups in the analysis of event-free survival, but no significant difference between total HDL-C groups and medium or small HDL subfraction groups. In particular, the multivariate Cox proportional hazards model showed that high HDL-L levels were associated with a lower risk of major adverse cardiovascular events (HR = 0.531, 95% CI 0.295–0.959), independent of potential confounders^37^.

The HDL-I subfractions (HDL-4 to 7) accounted for more than half of the total HDL-C level, with HDL-6 being the most predominant. Decreased levels of these subfractions have been associated with several cardiovascular events and risk factors based on literature data. Munchova and colleagues published a paper^53^ in which they examined the distribution of HDL subfractions in 27 mildly hypercholesterolemic and 21 healthy controls and found that HDL-6 (and HDL-7) concentrations were significantly lower in the patient group. Ezhov and colleagues found similar results^54^ when they studied the association between HDL subfractions and coronary atherosclerosis in 120 men. Their results showed significantly decreased levels of HDL-6 and -7 subfractions in patients with coronary lesions and found that the concentration of intermediate HDL subfractions (from HDL-4 to HDL-7) was inversely related to the presence and number of affected coronary arteries in middle-aged men. Femlak et al.^55^ have shown that HDL-6 and intermediate HDL subfractions are significantly lower in patients with advanced T2DM than in healthy or newly diagnosed patients.

Results of the present study that HDL-L levels show an inverse association with CVD risk furthermore, a decrease in HDL-L and a consequent increase in HDL-S is associated with elevated cardiovascular risk are consistent with the literature^37,42,56^.

Our results suggest that a decrease in HDL-6 and -7 subfractions may play a role in the high CVD risk among the Roma in general^40^. The question of whether this is due to the genetic predisposition identified in previous studies among the Roma^31,32,34^ or to other environmental and lifestyle factors associated with the Roma ethnic group^10–14^ requires further research.

Our current study has its strengths and limitations. On the one hand, the accurate identification of ethnicity is a common challenge for studies like ours. Due to the criteria of sample selection used in our present study (individuals were excluded by the investigation protocol), the sample populations cannot be interpreted as representative ones for the Hungarian general or Roma population. Given that the Hungarian general population may also include Roma individuals, the impact of ethnic differences estimated in the study may be underestimated. The cardiovascular risk assessment models used in the present study include only a limited number of traditional cardiovascular risk factors (age, gender, smoking, diabetic status, blood pressure, and cholesterol levels) and do not consider all known ones. This represents a major limitation when applying these equations to genetically susceptible people such as the Roma. Since the calculation formula of the risk estimation models includes HDL-C levels (as a protective/risk-reducing factor), our results slightly overestimate the risk-reducing and underestimate the risk-increasing effect of the identified subfractions. One of the major limitations of the present study is the small sample size which may result in limited statistical power. Although our results showing the association between CVD risk identified by different scoring calculations and HDL subfractions profile are statistically significant even after Bonferroni correction further analyses on larger sample populations of different ethnicities would be useful to confirm our conclusions.

On the other hand, the present study has several strengths. It is the first which compares the HDL subfraction profile of the Hungarian general and Roma populations and investigates whether there are differences between the two populations with the same HDL status. It also examines the association of HDL subfraction profile (in mmol/l and %) with cardiovascular risk factors. The present study is the first one to investigate the association between SCORE-estimated cardiovascular risk and HDL subfractions and to confirm the existence of a strong relationship between the SCORE- and FRS-estimated CVD risk and the HDL subfractions profile.

In conclusion, we have identified the significantly reduced levels of HDL subfractions 6 and 7 among the Roma. Furthermore, the present study is the first one to demonstrate a statistically significant association between HDL subfraction profile and cardiovascular risk estimated by SCORE and FRS. The results of the present research, which show that certain HDL subfractions are significantly reduced in individuals of Roma origin and that reduced levels of these subfractions are associated with increased cardiovascular risk, suggest that the distribution of HDL subfractions also contributes to the overall unfavorable cardiovascular risk among the Roma. Since genetic and lifestyle/environmental factors contribute to HDL-C levels approximately equally, one can raise the question of whether differences in the HDL subfraction profile of the Roma are influenced by their genetic inheritance or environmental and lifestyle factors, which requires further research.

## Supplementary Information

Supplementary Information 1.

Supplementary Information 2.

Supplementary Information 3.

Supplementary Information 4.

## Data Availability

The datasets generated and/or analyzed during the current study are not publicly available due to privacy/ethical restrictions but are available from the corresponding author on reasonable request.
