# Supplementary material for: The profile of HDL-C subfractions and their association with cardiovascular risk in the Hungarian general and Roma populations
Source: Sci Rep. 2022 Jun 28;12:10915. doi: 10.1038/s41598-022-15192-9 (PMC9240088; doi:10.1038/s41598-022-15192-9)
Supplement: Supplementary file 1 — Supplementary Information 1. [file 41598_2022_15192_MOESM1_ESM.docx]

**Supplementary Figure 1.** Composition of the HDL subfraction profile (in mmol/L and proportion in %) in the study populations by HDL-C status (normal and reduced).


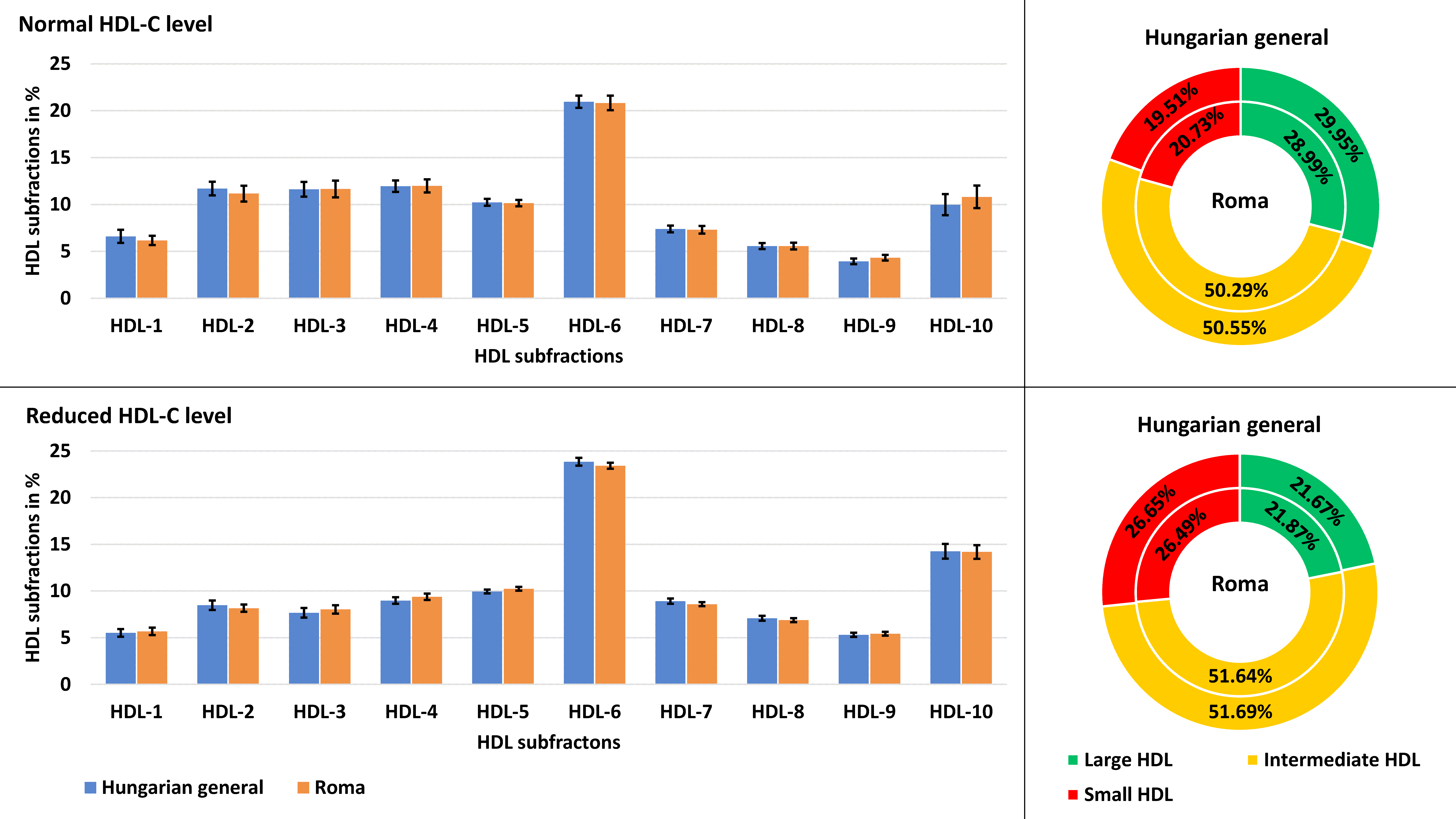

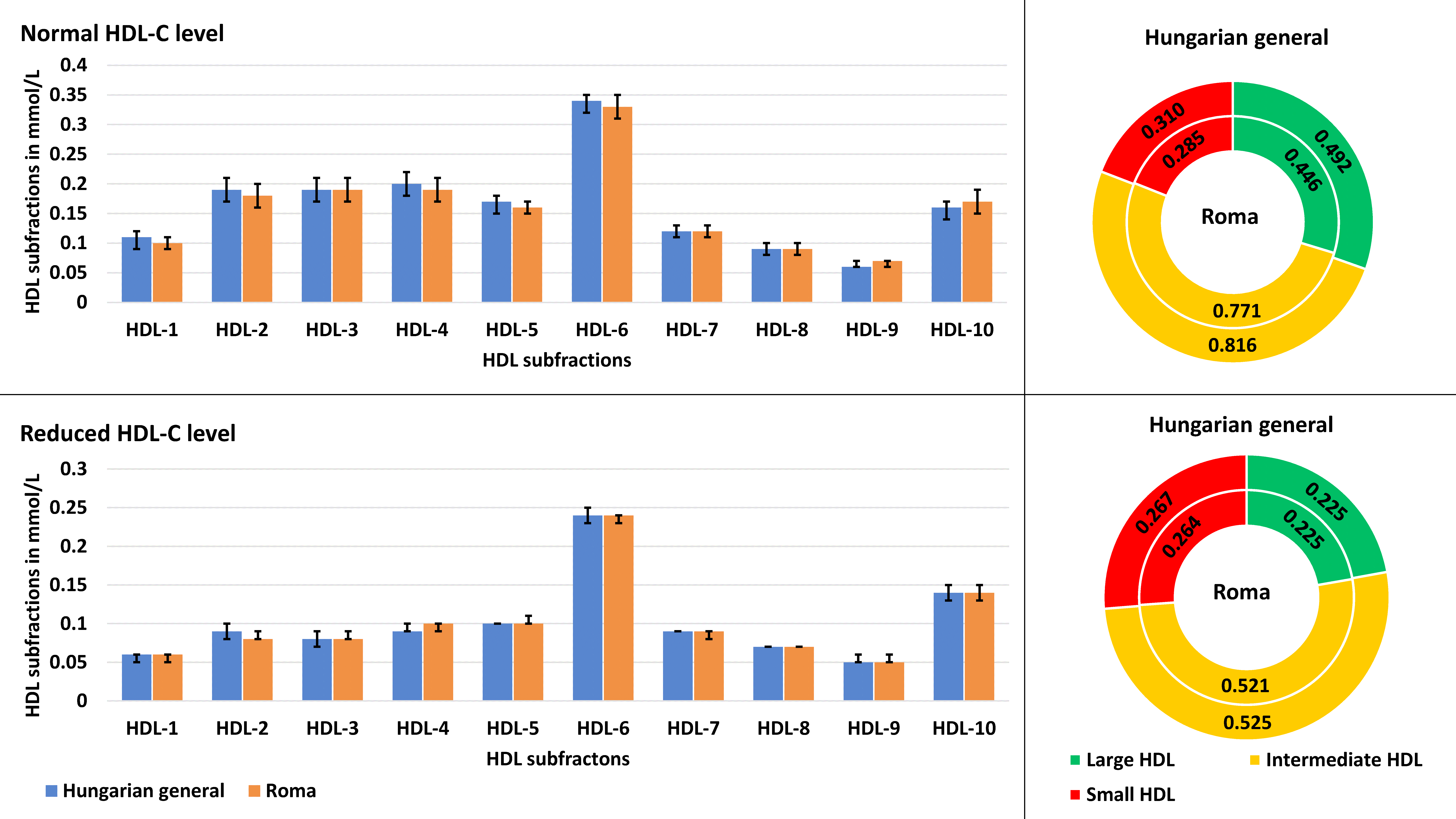


Large HDL: from HDL-1 to 3; intermediate HDL: from HDL-4 to 7; small HDL: from HDL-8 to 10. Threshold of significance after Bonferroni correction: p < 0.002.
