# Supplementary material for: The profile of HDL-C subfractions and their association with cardiovascular risk in the Hungarian general and Roma populations
Source: Sci Rep. 2022 Jun 28;12:10915. doi: 10.1038/s41598-022-15192-9 (PMC9240088; doi:10.1038/s41598-022-15192-9)
Supplement: Supplementary file 2 — Supplementary Information 2. [file 41598_2022_15192_MOESM2_ESM.docx]

**Supplementary Table 1.** Main characteristics of the study populations by factors used to estimate the cardiovascular risk in study populations by HDL-C status (normal vs. reduced HDL-C levels).

|  | Normal HDL-C levels | | | Reduced HDL-C levels | | |
| --- | --- | --- | --- | --- | --- | --- |
|  | Hungarian general  (n = 50) | Roma  (n = 50) | *p*-value | Hungarian general  (n = 115) | Roma  (n = 162) | *p*-value |
|  | Mean (95%CI) | |  | Mean (95%CI) | |  |
| Age (years) | 42.26  (38.57 - 45.95) | 42.18  (38.40 - 45.96) | 0.978 | 42.00  (39.85 - 44.15) | 39.53  (37.66 - 41.40) | 0.075 |
| BMI (kg/m^2^) | 25.03  23.80 - 26.25 | 23.32  21.67 - 24.96 | 0.027 | 28.24  27.19 - 29.28 | 29.24  28.25 - 30.22 | 0.215 |
| Systolic BP (mmHg) | 124.06  119.67 - 128.45 | 125.55  120.49 - 130.61 | 0.912 | 127.90  125.21 - 130.60 | 119.75  117.39 - 122.11 | <0.001* |
|  | Prevalence in % (95% CI) | | *p*-value | Prevalence in % (95% CI) | | *p*-value |
| Male | 50.00  (36.47 – 63.53) | 50.00  (36.47 – 63.53) | 0.999 | 36.52  (28.14 – 45.57) | 20.37  (14.72 – 27.06) | 0.003* |
| Female | 50.00  (36.47 – 63.53) | 50.00  (36.47 – 63.53) |  | 63.48  (54.43 – 71.86) | 79.63  (72.94 – 85.28) |  |
| Current smoker | 32.00  (20.36 – 45.66) | 62.22  (47.26 – 73.91) | 0.004* | 39.13  (30.57 – 48.23) | 70.19  (62.81 – 76.85) | <0.001* |
| Antihypertensive treatment | 16.00  (7.87 – 27.93) | 28.00  (17.04 – 41.42) | 0.148 | 27.83  (20.26 – 36.49 | 27.16  (20.76 – 34.37) | 0.903 |
| Diabetes | 6.67  (1.91 – 16.73) | 16.22  (7.06 - 30.41) | 0.169 | 7.41  (3.56 – 13.49) | 14.73  (9.42 – 21.60) | 0.077 |

95% CI: 95% confidence interval; BMI: body mass index; BP: blood pressure. *: significant results after test correction (*p* < 0.007).
