# Supplementary material for: The profile of HDL-C subfractions and their association with cardiovascular risk in the Hungarian general and Roma populations
Source: Sci Rep. 2022 Jun 28;12:10915. doi: 10.1038/s41598-022-15192-9 (PMC9240088; doi:10.1038/s41598-022-15192-9)
Supplement: Supplementary file 3 — Supplementary Information 3. [file 41598_2022_15192_MOESM3_ESM.docx]

**Supplementary Table 2.** The average value of HDL subfractions (in mmol/L) in the CVD risk categories by Systematic COronary Risk Evaluation and Framingham Risk Scores and the results of p for trend analyses.

|  | Systematic COronary Risk Evaluation | | | | Framingham Risk Scores | | | | | | | |
| --- | --- | --- | --- | --- | --- | --- | --- | --- | --- | --- | --- | --- |
|  | High-risk algorithm | | | | CHD | | | | CVD in generally | | | |
|  | Low risk (<2%) | Intermediate risk (2-<5%) | High risk (≥5%) | p for trend | Low risk (<10%) | Intermediate risk (10-20%) | High risk (>20%) | p for trend | Low risk (<10%) | Intermediate risk (10-20%) | High risk (>20%) | p for trend |
| HDL-1 | 0.071 | 0.071 | 0.054 | 0.019 | 0.071 | 0.045 | 0.036 | <0.001* | 0.073 | 0.061 | 0.048 | <0.001* |
| HDL-2 | 0.119 | 0.131 | 0.087 | 0.037 | 0.117 | 0.079 | 0.055 | <0.001* | 0.117 | 0.107 | 0.087 | 0.007 |
| HDL-3 | 0.118 | 0.128 | 0.082 | 0.011 | 0.114 | 0.074 | 0.045 | <0.001* | 0.115 | 0.103 | 0.081 | 0.002* |
| HDL-4 | 0.128 | 0.135 | 0.096 | 0.032 | 0.123 | 0.091 | 0.061 | <0.001* | 0.124 | 0.116 | 0.093 | 0.003* |
| HDL-5 | 0.124 | 0.124 | 0.101 | 0.033 | 0.119 | 0.102 | 0.079 | 0.006 | 0.121 | 0.113 | 0.098 | 0.004 |
| HDL-6 | 0.282 | 0.268 | 0.237 | 0.007 | 0.267 | 0.241 | 0.198 | 0.010 | 0.271 | 0.256 | 0.228 | 0.008 |
| HDL-7 | 0.104 | 0.097 | 0.091 | 0.026 | 0.098 | 0.092 | 0.078 | 0.206 | 0.099 | 0.094 | 0.086 | 0.081 |
| HDL-8 | 0.082 | 0.077 | 0.073 | 0.061 | 0.077 | 0.074 | 0.065 | 0.529 | 0.078 | 0.075 | 0.069 | 0.289 |
| HDL-9 | 0.061 | 0.058 | 0.057 | 0.326 | 0.058 | 0.059 | 0.054 | 0.598 | 0.058 | 0.059 | 0.054 | 0.792 |
| HDL-10 | 0.151 | 0.143 | 0.154 | 0.908 | 0.145 | 0.158 | 0.161 | 0.205 | 0.145 | 0.151 | 0.149 | 0.360 |
| HDL-L | 0.308 | 0.330 | 0.223 | 0.016 | 0.301 | 0.199 | 0.136 | <0.001* | 0.305 | 0.271 | 0.216 | 0.001* |
| HDL-I | 0.639 | 0.624 | 0.524 | 0.016 | 0.608 | 0.526 | 0.416 | 0.002* | 0.616 | 0.579 | 0.505 | 0.003* |
| HDL-S | 0.294 | 0.278 | 0.284 | 0.468 | 0.280 | 0.291 | 0.281 | 0.393 | 0.281 | 0.285 | 0.272 | 0.725 |

HDL-L: large HDL (from HDL-1 to 3); HDL-I: intermediate HDL (from HDL-4 to 7); HDL-S: small HDL (from HDL-8 to 10); CVD: cardiovascular diseases; CHD: coronary heart disease. *: significant results after test correction (*p* < 0.004).
