# Supplementary material for: The profile of HDL-C subfractions and their association with cardiovascular risk in the Hungarian general and Roma populations
Source: Sci Rep. 2022 Jun 28;12:10915. doi: 10.1038/s41598-022-15192-9 (PMC9240088; doi:10.1038/s41598-022-15192-9)
Supplement: Supplementary file 4 — Supplementary Information 4. [file 41598_2022_15192_MOESM4_ESM.docx]

**Supplementary Table 3.** The average proportion of HDL subfractions (in %) in the CVD risk categories by Systematic COronary Risk Evaluation and Framingham Risk Scores and the results of p for trend analyses.

|  | Systematic COronary Risk Evaluation | | | | Framingham Risk Scores | | | | | | | |
| --- | --- | --- | --- | --- | --- | --- | --- | --- | --- | --- | --- | --- |
|  | High-risk algorithm | | | | CHD | | | | CVD in generally | | | |
|  | Low risk (<2%) | Intermediate risk (2-<5%) | High risk (≥5%) | p for trend | Low risk (<10%) | Intermediate risk (10-20%) | High risk (>20%) | p for trend | Low risk (<10%) | Intermediate risk (10-20%) | High risk (>20%) | p for trend |
| HDL-1 | 5.687 | 5.585 | 5.035 | 0.190 | 5.824 | 4.376 | 4.467 | 0.001* | 5.928 | 5.239 | 4.746 | 0.001* |
| HDL-2 | 9.299 | 10.015 | 8.159 | 0.430 | 9.349 | 7.580 | 6.433 | 0.002* | 9.337 | 8.872 | 8.363 | 0.123 |
| HDL-3 | 9.079 | 9.637 | 7.568 | 0.068 | 8.975 | 7.088 | 5.267 | 0.001* | 9.026 | 8.305 | 7.750 | 0.017 |
| HDL-4 | 9.922 | 10.514 | 9.071 | 0.293 | 9.977 | 8.848 | 7.333 | 0.008 | 9.971 | 9.719 | 9.125 | 0.097 |
| HDL-5 | 10.027 | 10.186 | 9.800 | 0.731 | 10.075 | 10.056 | 9.400 | 0.515 | 10.087 | 10.107 | 9.804 | 0.249 |
| HDL-6 | 23.039 | 22.417 | 23.200 | 0.816 | 22.907 | 23.876 | 23.467 | 0.071 | 22.931 | 23.126 | 23.288 | 0.273 |
| HDL-7 | 8.509 | 8.122 | 8.879 | 0.837 | 8.412 | 9.064 | 9.233 | 0.046 | 8.423 | 8.518 | 8.858 | 0.288 |
| HDL-8 | 6.713 | 6.417 | 7.168 | 0.537 | 6.633 | 7.368 | 7.867 | 0.011 | 6.630 | 6.823 | 7.125 | 0.146 |
| HDL-9 | 5.068 | 4.854 | 5.662 | 0.161 | 5.017 | 5.876 | 6.533 | <0.001* | 4.977 | 5.365 | 5.579 | 0.007 |
| HDL-10 | 12.666 | 12.246 | 15.444 | 0.051 | 12.835 | 15.868 | 19.967 | 0.002* | 12.698 | 13.921 | 15.350 | 0.005 |
| HDL-L | 24.059 | 25.239 | 20.771 | 0.181 | 24.147 | 19.044 | 16.167 | <0.001* | 24.291 | 22.414 | 20.863 | 0.010 |
| HDL-I | 51.498 | 51.242 | 50.947 | 0.152 | 51.370 | 51.844 | 49.467 | 0.999 | 51.412 | 51.467 | 51.079 | 0.701 |
| HDL-S | 24.447 | 23.520 | 28.291 | 0.077 | 24.488 | 29.124 | 34.367 | 0.001* | 24.304 | 26.125 | 28.058 | 0.006 |

HDL-L: large HDL (from HDL-1 to 3); HDL-I: intermediate HDL (from HDL-4 to 7); HDL-S: small HDL (from HDL-8 to 10); CVD: cardiovascular diseases; CHD: coronary heart disease. *: significant results after test correction (*p* < 0.004).
